# Dural Convexity Chondroma Mimicking Meningioma in a Young Female

**DOI:** 10.7759/cureus.20715

**Published:** 2021-12-26

**Authors:** Danyas Sarathy, Matthew H Snyder, Leonel Ampie, Debra Berry, Hasan R Syed

**Affiliations:** 1 Department of Neurological Surgery, University of Virginia, Charlottesville, USA; 2 Department of Neurological Surgery, Tufts Medical Center, Boston, USA; 3 Department of Pathology, University of Virginia, Charlottesville, USA; 4 Department of Neurological Surgery, Children's National Hospital, Washington DC, USA

**Keywords:** intracranial chondroma, neuro-surgery, brain tumors cns tumors, neurology and neuro-oncology, head and neck tumors

## Abstract

Intracranial meningeal convexity chondroma is a rare benign lesion hypothesized to stem from remnant chondrocyte precursors of embryonic origin. This lesion often masquerades as meningioma given the similar dural-based attachment and pattern of calcification. We describe the case of a 26-year-old female with incidentally discovered convexity meningeal chondroma, originally presumed to be a meningioma. In this case, we share our diagnostic and operative intervention and outcome and discuss the unique pathologic findings in this lesion that differentiate it from similar appearing lesions. To the authors’ knowledge, there are fewer than 20 cases of convexity meningeal chondroma in the literature; thus, we also provide a brief review of the literature regarding this rare pathology.

## Introduction

Chondromas are rare and indolent tumors originating from mature chondrocytes producing cartilaginous matrix [1]. They represent benign neoplasms and are most commonly present in smaller bones (e.g., hands/feet) and less commonly in larger bones (e.g., femur/humerus) [2]. On the other hand, intracranial chondromas are exceedingly rare, comprising less than 0.5% of all intracranial neoplasms [3]. This pathologic entity was originally discussed in 1851 by Hirschfeld, followed by the first operative resection of this neoplasm by Nixon in 1982 [4].

Intracranial chondromas most often present extradural to the skull base, in either a sellar or parasellar location [5]. Chondroma arising from the dura is rarer, with few other cases described in the literature.

We present the case of a 26-year-old with an incidentally discovered left frontal convexity lesion initially concerning for meningioma, and later histologically found to be a chondroma. We seek to share our diagnostic approach, surgical management, and operative outcome of this patient in order to add to the paucity of literature available on this rare neoplasm.

## Case presentation

**Figure 1:**
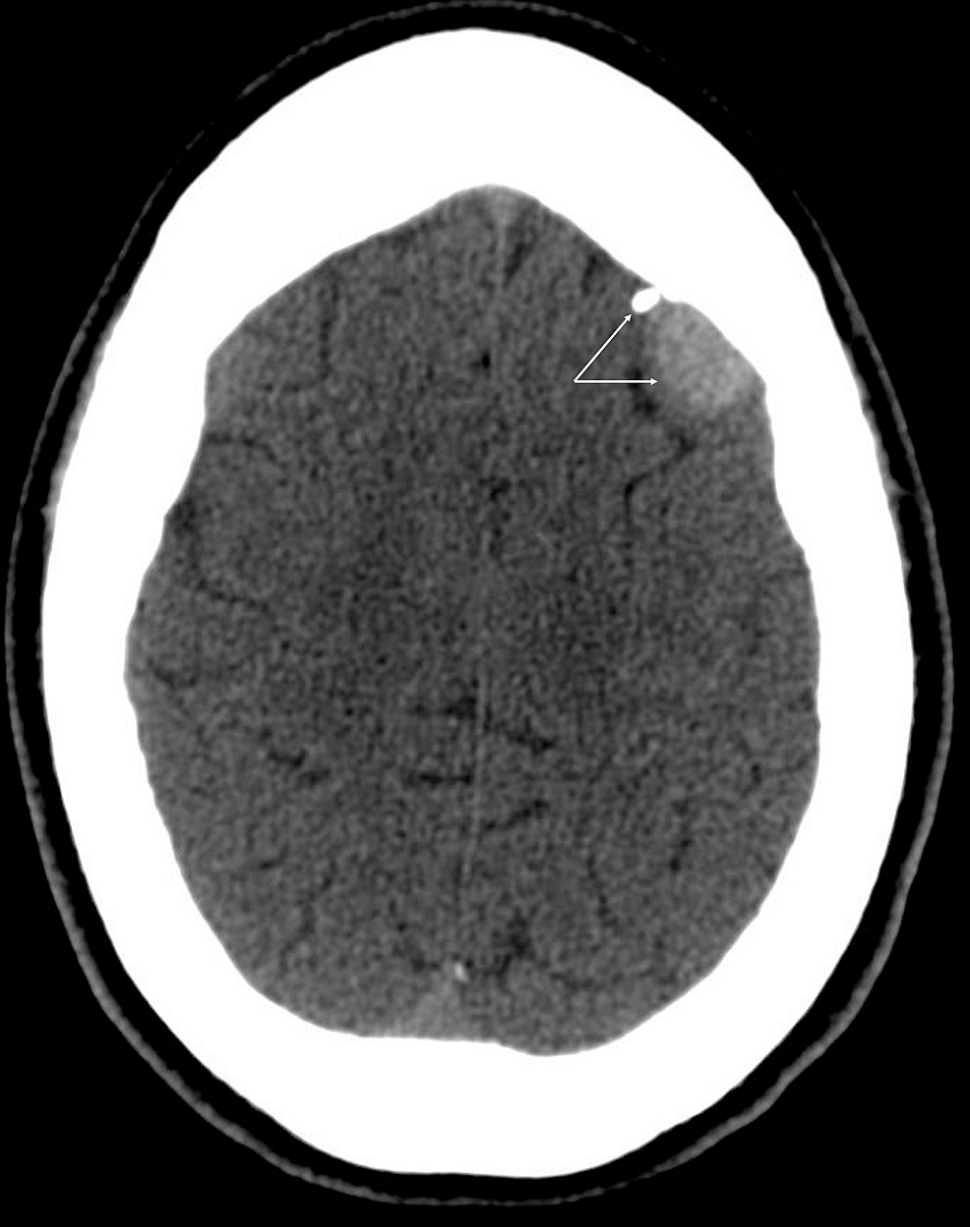
Initial CTH demonstrating the lesion. Non-contrasted CTH with axial view demonstrating a round hyper-dense extra-axial lesion in the left frontal cortex; there is an associated calcium deposit suggestive of calcified meningioma (arrows). Sagittal and coronal sections are not shown due to poor image fidelity impeding MPR. CTH: CT scan of the head; MPR: Multiplanar reformation.

The patient is a 26-year-old female with poorly controlled type II diabetes and Crohn's disease who initially presented to her primary care physician (PCP) with vague neurological symptoms of dizziness, headache, and memory loss one week following a motor vehicle collision. The patient also mentioned symptoms resembling absence-type seizures. Her PCP ordered a CT scan of the head (CTH) to assess for intracranial pathology, which demonstrated a left frontal hyperdense extra-axial lesion with minimal surrounding edema, suggestive of a meningioma. Calcification present within the lesion suggested an indolent, long-standing mass (Figure 1).

**Figure 2:**
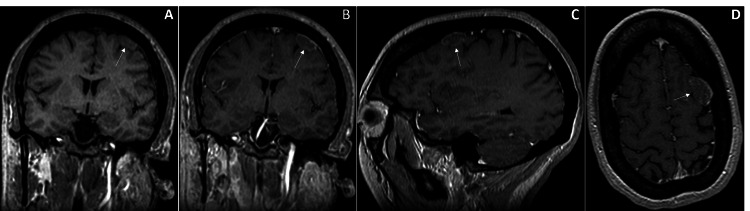
MRI brain further characterizing the left frontal lesion. Pre-contrasted coronal T1 section demonstrating a well-marginated, iso-dense, dural-based, extra-axial round mass lesion along left frontal convexity (A). Post-contrasted coronal (B), sagittal (C), and axial (D) T1 sections re-demonstrating the same left convexity lesion, now with peripheral contrast enhancement.

Subsequent MRI brain re-demonstrated the dural-based extra-axial mass lesion along the left frontal convexity, but with less enhancement as expected with a meningioma. The lesion measured 15 x 25 mm in the axial plane (Figure 2).

**Figure 3:**
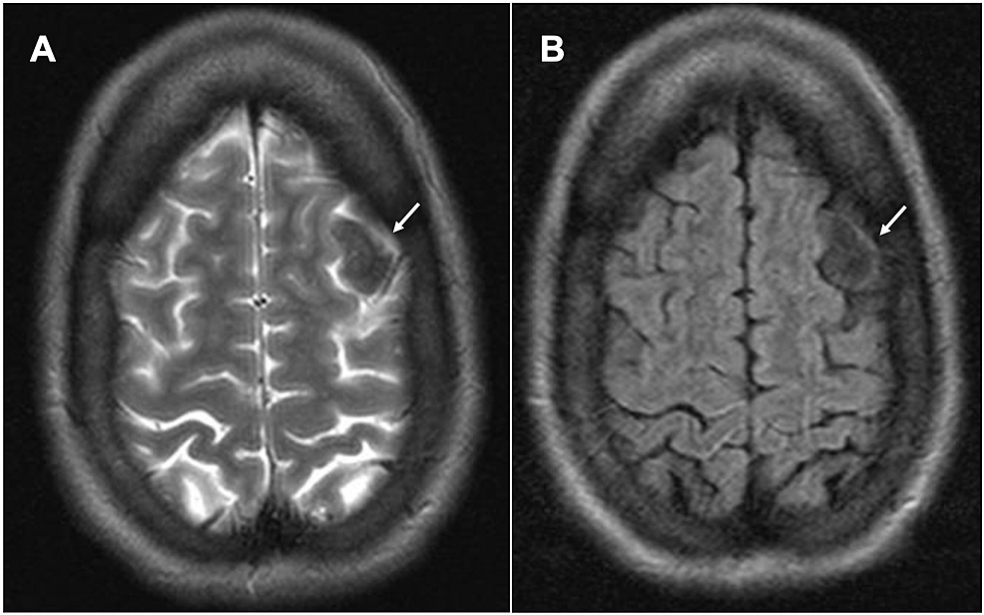
MRI brain demonstrating lack of T2 or FLAIR signal changes. Axial T2 section re-demonstrating the left frontal convexity mass as a hypo-intense region without adjacent CSF signal changes (A). Axial FLAIR section demonstrating the same lesion, and without adjacent signal changes concerning surrounding pathologic edema. FLAIR: Fluid-attenuated inversion recovery; CSF: Cerebrospinal fluid.

Of note, there were no T2 or fluid-attenuated inversion recovery (FLAIR) signal changes around the lesion, reducing the concern for pathologic edema (Figure 3).

She was referred to our institution for further diagnostic workup. Her neurological examination was non-focal and unremarkable for any signs of increased intracranial pressure. Repeat MRI three months later showed no change of the lesion. The patient continued to have symptoms likely attributable to her recent concussion. However, given the atypical imaging features of the left frontal convexity lesion that were not wholly consistent with meningioma, surgical intervention was offered to both definitively diagnose and treat the lesion. At this time, craniotomy with intraoperative neuro-navigation was planned to excise the lesion, as her blood glucose was slightly under control over this interval.

**Figure 4:**
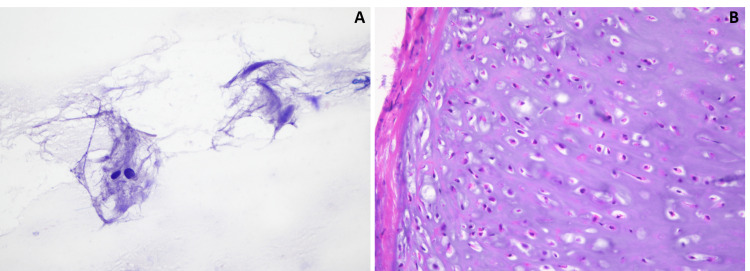
Intraoperative frozen smear and section. Intraoperative frozen smear and section demonstrate abundant cartilaginous material with scattered, bland cells (A), and mature cartilage without atypical features (B).

**Figure 5:**
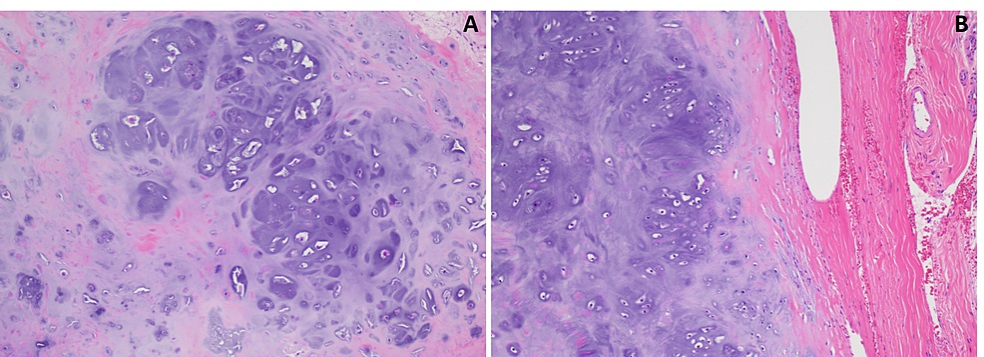
Permanent histological sections. Permanent H&E sections reveal a well-circumscribed neoplasm of benign hyaline cartilage (A), which appears to arise from the dura mater (B).

The patient underwent a left frontal craniotomy with a partial pterional incision. The tumor was found to extend further anteriorly than suggested by neuro-navigation. The dura surrounding the tumor was excised circumferentially with a clean margin. The tumor was found to reflect off the cortical surface with no evidence of adhesion, and gross total resection was achieved. An intraoperative frozen section histologically revealed atypical mature cartilage, and abundant cartilaginous material with scattered, bland cells, inconsistent with a meningioma (Figure 4). The permanent section elucidated the final diagnosis of meningeal chondroma, with sections detailing a well-circumscribed neoplasm of benign hyaline cartilage arising from the dura (Figure 5).

Her postoperative course was mostly uneventful, and while she was discharged on her fourth postoperative day, she returned for admission ten days later complaining of a frontal headache. She was discharged the next day after the resolution of her symptoms, and an unremarkable MRI revealing no infection, hemorrhage, or hydrocephalus. No adjuvant therapy was performed. At one year follow-up, the patient was free of any recurrence and doing well. She has not required follow-up visits since then.

## Discussion

The etiology of chondroma is proposed to stem from the remnants of embryonic chondrocyte precursors that are housed within synchondroses, as these are joints composed entirely of hyaline cartilage. This leads to the most common location of intracranial chondroma being the skull base, specifically along the spheno-petrosal, spheno-occipital, and petro-clival synchondroses, this is where the majority of remnant chondrocytes would be found [5,6]. Another etiologic explanation for chondroma includes metaplasia of fibroblasts, which leads to reports of chondroma arising from paranasal sinuses with intracranial extension and even intraventricular locations [2,7,8]. There are few other case reports of convexity meningeal chondroma in the literature [2,9-17], demonstrating that while dural attachment of a chondroma is quite rare, it is an essential diagnostic possibility to consider.

Given the rare nature of intracranial chondroma, few studies have looked specifically at the unique radiographic features of this tumor. However, a case series of six patients by Duan F et al. describes that all their cases presented as a well-demarcated lesion with irregular lobulation, variable density, and calcification on CT. Calcification is explained by the slow-growing and indolent course of this tumor. It is likely to be more present in chondroma than in meningioma, where it occurs in approximately 25% of cases, although more data is needed to support this fully [18,19]. Duan et al. further classify the MR imaging characteristics of this tumor as a heterogeneously enhancing, hypointense lesion on T1-weighted imaging, and mixed hyperintense/hypointense on T2 imaging. On the other hand, meningioma tends to show more homogenous enhancement on both T1 and T2 weighted imaging [19,20]. As noted in our case, the lesion was found to have imaging characteristics that were atypical for meningioma, favoring an alternative diagnosis (Figure 2).

Tissue diagnosis is the only reliable method to distinguish intracranial chondroma from other mimics, like meningioma. The literature consistently describes chondroma as comprising bland chondrocytes in a hyaline cartilage matrix [7,12,16,18]. Positive staining for vimentin and S-100 protein, with negative staining for cytokeratin (CK) and epithelial membrane antigen (EMA), helps differentiate chondroma from chordoma. Immunohistochemical staining between chondroma and chondrosarcoma is similar, but other features such as nuclear and morphological atypia help to distinguish these two neoplasms [6].

Because intracranial chondroma is a benign and slow-growing tumor, resection alone is usually sufficient, although occasionally adjuvant radiotherapy is required. However, there are no outcomes data specifically on meningeal chondroma, a case series of 66 patients with intracranial chondroma by Weng JC et al. details the difference in outcome between total vs. partial resection and the use of adjuvant radiotherapy (which was employed in cases with partial resection and atypical histologic findings). In their cohort, overall survival was significantly improved with gross total resection as compared to partial resection, but not subtotal resection. Factors significantly associated with greater progression-free survival included tumor size <3.1 cm, age >33 years, histology without atypia/mitotic activity, and surgery with adjuvant radiation therapy [6]. In our case, gross total resection was possible and there was no need for adjuvant radiotherapy. Given the small size of the tumor and our ability to achieve gross total resection, our patient was expected to have a good outcome.

## Conclusions

Intracranial chondroma is a rare, indolent tumor that infrequently presents as a convexity lesion with dural attachment. This case report highlights the key clinical features of this rare pathology. The clinical presentation of this tumor is variable, but in most cases, will be a slow-growing, surreptitious mass of the skull base. Diagnosis of this lesion is a multimodal effort, with key radiographic findings including heterogeneous enhancement and density on CT, and mixed hyper- and hypo-intensity on T2 MRI. This lesion can be distinguished from meningioma, which tends to have more homogenous enhancement on T1- and T2-weighted imaging, even though both lesions may show calcification and the appearance of dural tails. Histologic diagnosis is definitive, as chondroma will show characteristic chondrocytes in a matrix of benign hyaline cartilage. 

With respect to clinical relevance, care must be taken while distinguishing the more common lesion of meningioma from convexity chondroma. This may occasionally be difficult due to similar (but not identical) radiographic findings as delineated earlier. The treatment of choice when possible (e.g., location of the lesion away from the eloquent brain) is total resection of the mass. When achieved, favorable outcomes are generally the rule. Should total resection not be amenable, or atypical histology found, adjuvant radiotherapy is a useful strategy to consider.

## References

[1] Suster D, Hung YP, Nielsen GP (2020). Differential diagnosis of cartilaginous lesions of bone. Arch Pathol Lab Med.

[2] Reinshagen C, Redjal N, Sajed DP, Nahed BV, Walcott BP (2016). Intracranial dural based chondroma. J Clin Neurosci.

[3] Agrawal R, Saroha A (2019). Intracranial chondroma of the falx cerebri: a rare case report with review of literature. Asian J Neurosurg.

[4] Maheshwari V, Mehdi G, Varshney M, Jain A, Vashishtha S, Gaur K, Srivastava VK (2011). Intracranial chondroma: a rare entity. BMJ Case Rep.

[5] Pospiech J, Mehdorn HM, Reinhardt V, Grote W (1989). Sellar chondroma in a case of Ollier's disease. Neurochirurgia (Stuttg).

[6] Weng JC, Li D, Li H (2017). Surgical management and outcomes of intracranial chondromas: a single-center case series of 66 patients. World Neurosurg.

[7] Nikoobakht M, Shojaei H, Baseri YK, Shojaei SF (2020). An intraventricular type of chondroma: a case report. Turk Neurosurg.

[8] Zabolotnii DI, Palamar OI, Guk AP, Zinchenko DA, Gorbach ON (2013). [Bone and cartilaginous tumours in the sino-paranasal region with intracranial extension. Peculiarities of surgical treatment]. [Russian]. Vestn Otorinolaringol.

[9] Abeloos L, Maris C, Salmon I, Balériaux D, Sadeghi N, Lefranc F (2012). Chondroma of the dural convexity: a case report and literature review. Neuropathology.

[10] Erdogan S, Zorludemir S, Erman T, Akgul E, Ergin M, Ildan F, Bagdatoglu H (2006). Chondromas of the falx cerebri and dural convexity: report of two cases and review of the literature. J Neurooncol.

[11] Colpan E, Attar A, Erekul S, Arasil E (2003). Convexity dural chondroma: a case report and review of the literature. J Clin Neurosci.

[12] Nakayama M, Nagayama T, Hirano H, Oyoshi T, Kuratsu J (2001). Giant chondroma arising from the dura mater of the convexity. Case report and review of the literature. J Neurosurg.

[13] Feierabend D, Maksoud S, Lawson McLean A, Koch A, Kalff R, Walter J (2018). Giant convexity chondroma with meningeal attachment. Clin Neurol Neurosurg.

[14] Atalay FO, Ozgun G, Tolunay S, Bekar A (2014). Intracranial extra-axial chondroma: a case report. J Nippon Med Sch.

[15] Cosar M, Iplikcioglu AC, Bek S, Gokduman CA (2005). Intracranial falcine and convexity chondromas: two case reports. Br J Neurosurg.

[16] Delgado-Lopez PD, Martin-Velasco V, Galacho-Harriero AM, Castilla-Diez JM, Rodriguez-Salazar A, Echevarria-Iturbe C (2007). Large chondroma of the dural convexity in a patient with Noonan’s syndrome. Case report and review of the literature. Neurocirugia (Astur).

[17] Nakazawa T, Inoue T, Suzuki F, Nakasu S, Handa J (1993). Solitary intracranial chondroma of the convexity dura: case report. Surg Neurol.

[18] Lyndon D, Lansley JA, Evanson J, Krishnan AS (2019). Dural masses: meningiomas and their mimics. Insights Imaging.

[19] Duan F, Qiu S, Jiang J (2012). Characteristic CT and MRI findings of intracranial chondroma. Acta Radiol.

[20] Buetow MP, Buetow PC, Smirniotopoulos JG (1991). Typical, atypical, and misleading features in meningioma. Radiographics.

